# Di-*tert*-butyl 2,2′-[(2-hydroxy­ethyl)aza­nedi­yl]diacetate

**DOI:** 10.1107/S1600536809049022

**Published:** 2009-11-21

**Authors:** Yang Yang, Lin Zhu, Huabei Zhang

**Affiliations:** a Key Laboratory of Radiopharmaceuticals, Ministry of Education, Department of Chemistry, Beijing Normal University, Xin Jie Kou Wai Street 19, 100875 Beijing, People’s Republic of China

## Abstract

In the title compound, C_14_H_27_NO_5_, the hydr­oxy group and one of the acetate carbonyl O atoms are linked by an intra­molecular O—H⋯O hydrogen bond, forming an eight-membered ring. This inter­action gives rise to an asymmetric mol­ecular conformation.

## Related literature

For details of the synthesis, see: Williams & Rapoport (1993 ▶); Amedio *et al.* (2000 ▶). For possible applications of the title compound, see: Yang *et al.* (2007 ▶).
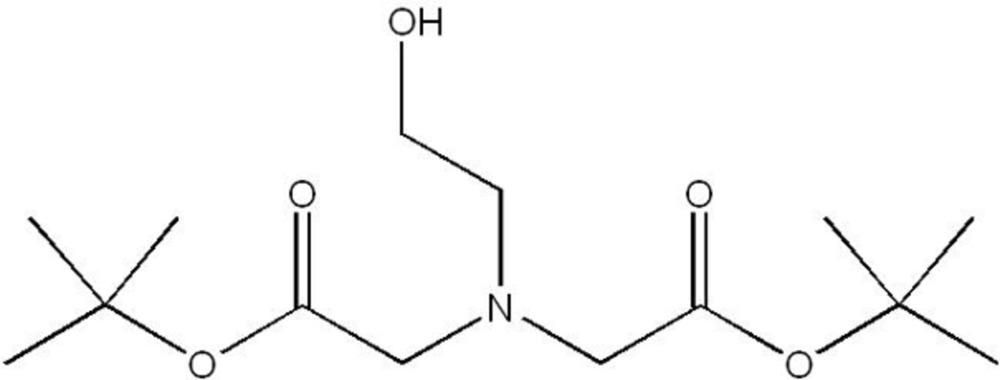


## Experimental

### 

#### Crystal data

C_14_H_27_NO_5_
*M* *_r_* = 289.37
Orthorhombic, 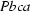

*a* = 11.9767 (4) Å
*b* = 9.7187 (3) Å
*c* = 29.3476 (7) Å
*V* = 3416.00 (18) Å^3^
*Z* = 8
Mo *K*α radiation
μ = 0.08 mm^−1^
*T* = 296 K
0.36 × 0.21 × 0.08 mm

#### Data collection

Bruker SMART APEX CCD area-detector diffractometer
Absorption correction: multi-scan (*SADABS*; Bruker, 2007 ▶) *T*_min_ = 0.970, *T*_max_ = 0.993
12339 measured reflections
3958 independent reflections
2503 reflections with *I* > 2σ(*I*)
*R*_int_ = 0.022

#### Refinement

*R*[*F* ^2^ > 2σ(*F* ^2^)] = 0.046
*wR*(*F* ^2^) = 0.128
*S* = 1.02
3958 reflections
190 parameters
1 restraint
H atoms treated by a mixture of independent and constrained refinement
Δρ_max_ = 0.13 e Å^−3^
Δρ_min_ = −0.17 e Å^−3^

### 

Data collection: *SMART* (Bruker, 1998 ▶); cell refinement: *SAINT* (Bruker, 2005 ▶); data reduction: *SAINT*; program(s) used to solve structure: *SHELXTL* (Sheldrick, 2008 ▶); program(s) used to refine structure: *SHELXTL*; molecular graphics: *SHELXTL*; software used to prepare material for publication: *SHELXTL*.

## Supplementary Material

Crystal structure: contains datablocks I, global. DOI: 10.1107/S1600536809049022/gk2237sup1.cif

Structure factors: contains datablocks I. DOI: 10.1107/S1600536809049022/gk2237Isup2.hkl

Additional supplementary materials:  crystallographic information; 3D view; checkCIF report

## Figures and Tables

**Table 1:** Hydrogen-bond geometry (Å, °)

*D*—H⋯*A*	*D*—H	H⋯*A*	*D*⋯*A*	*D*—H⋯*A*
O5—H5⋯O2	0.848 (10)	2.128 (17)	2.8658 (18)	145 (2)

**Fig. 1.**
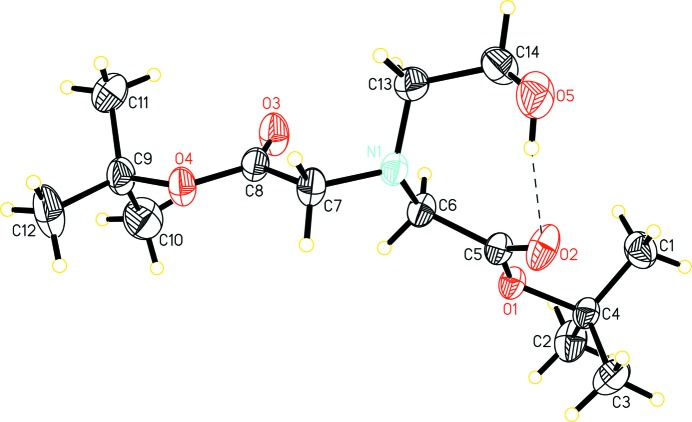
The molecule the title compound, showing the atomic numbering; the displacement ellipsoids were drawn at the 30% probability level.

**Fig. 2.**
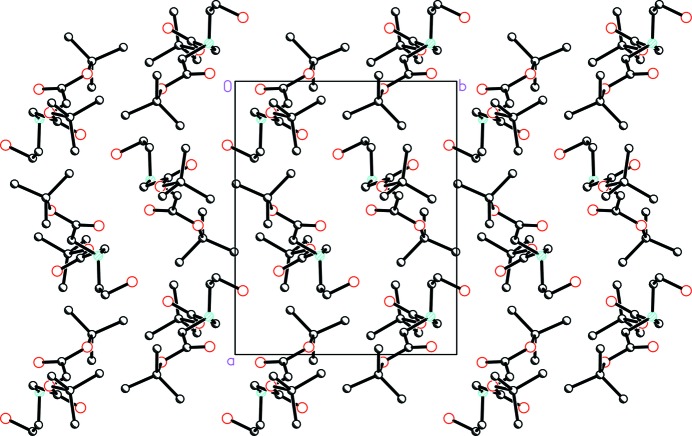
The packing diagram of title compound (for clarity, all H atoms are not shown).

## supplementary crystallographic information

### Comment

The aminodiacetate derivatives can find potential application when labeled by the novel ^99^*^m^*Tc(CO)_2_(NO)^2+^ core to explore the ^99^*^m^*Tc labelled radiopharmaceuticals (Yang *et al.*, 2007). Thus, the development of aminodiacetate derivatives may lead to obtain some new imaging agents labelled by ^99^*^m^*Tc core. Here we report the crystal structure of the title compound which can be used as a precursor in the synthesis of aminodiacetate derivatives.

The molecule of the title compound is shown in Fig. 1. The molecular conformation is to a large extentd determined by the intramolecular hydrogen bond O(5)—H(5)···O(2) (Table 1) which is a part of the eight-membered ring –O(5)—C(14)—C(13)—N(1)—C(6)—C(5)—O(2)······H(5)-. In the above ring, the torsion angles N(1)—C(13)—C(14)—O(5) and N(1)—C(6)—C(5)—O(2) are -57.2 (2)° and -3.5 (2)°.

### Experimental

Tert-butyl 2-bromoacetate (22 g, 114 mmol) and KHCO_3_ (13 g, 130 mmol) were dissolved in DMF (100 ml) at 0°C. Then 2-aminoethanol (3.2 ml, 50 mmol) was added to the solution in drops within 1 h. After adding 2-aminoethanol, the solution was heated at 55 °C for 20 h. Subsequently, the mixture was washed by the saturated NaHCO_3_ solution and the crude product was extracted by ethyl acetate. After that, the organic phase was washed by saturated NaCl solution and the new organic phase was then dried by Na_2_SO_4_ for 48 h. After filtering the solution, the crude product was obtained. The crude product was recrystallized from ethyl acetate giving colorless block crystals of the title compound suitable for the single-crystal X-ray diffraction. IRnfrared Spectrum: 3438.3 cm^-1^; 2978.5 cm^-1^; 2933.7 cm^-1^; 1456.8 cm^-1^; 1393.6 cm^-1^; 1732.0 cm^-1^; 1368.6 cm^-1^; 1223.5 cm^-1^; 1070.9 cm^-1^; 1150.5 cm^-1. 1^H-NMR (CDCl_3_, 400 MHz): 3.90 (s, 1H), 3.53 (t, J = 5.1 Hz, 2H), 3.45 (s, 4H), 2.89 (t, J = 5.1 Hz, 2H), 1.47 (s, 18H). ^13^C-NMR (CDCl_3_, 400 MHz): δ 28.13, 56.64, 57.07, 59.34, 81.49, 171.46. MS:m/*z* 290.3 [*M* + H].

### Refinement

The H atoms bound to C atoms were introduced in idealized positions (C-H = 0.96-0.97 Å) and allowed to ride on their respective parent atoms with *U*_iso_(H) =1.2 *U*_eq_(C). The H atom from the hydroxy group was located in a difference Fourier synthesis and in the refinement the O-H distance was restrained to 0.86 (1) Å [*U*_iso_(H) =1.5 *U*_eq_(O)].

### Crystal data

**Table:**

C_14_H_27_NO_5_	*F*(000) = 1264
*M**_r_* = 289.37	*D*_x_ = 1.125 Mg m^−^^3^
Orthorhombic, *P**b**c**a*	Mo *K*α radiation, λ = 0.71073 Å
Hall symbol: -P 2ac 2ab	Cell parameters from 3711 reflections
*a* = 11.9767 (4) Å	θ = 2.2–26.1°
*b* = 9.7187 (3) Å	µ = 0.08 mm^−^^1^
*c* = 29.3476 (7) Å	*T* = 296 K
*V* = 3416.00 (18) Å^3^	Block, colorless
*Z* = 8	0.36 × 0.21 × 0.08 mm

### Data collection

**Table:**

Bruker SMART APEX CCD area-detector diffractometer	3958 independent reflections
Radiation source: fine-focus sealed tube	2503 reflections with *I* > 2σ(*I*)
graphite	*R*_int_ = 0.022
phi and ω scans	θ_max_ = 27.6°, θ_min_ = 2.2°
Absorption correction: multi-scan (*SADABS*; Bruker, 2007)	*h* = −15→10
*T*_min_ = 0.970, *T*_max_ = 0.993	*k* = −12→7
12339 measured reflections	*l* = −38→25

### Refinement

**Table:**

Refinement on *F*^2^	Primary atom site location: structure-invariant direct methods
Least-squares matrix: full	Secondary atom site location: difference Fourier map
*R*[*F*^2^ > 2σ(*F*^2^)] = 0.046	Hydrogen site location: inferred from neighbouring sites
*wR*(*F*^2^) = 0.128	H atoms treated by a mixture of independent and constrained refinement
*S* = 1.02	*w* = 1/[σ^2^(*F*_o_^2^) + (0.0551*P*)^2^ + 0.5511*P*] where *P* = (*F*_o_^2^ + 2*F*_c_^2^)/3
3958 reflections	(Δ/σ)_max_ = 0.001
190 parameters	Δρ_max_ = 0.13 e Å^−^^3^
1 restraint	Δρ_min_ = −0.17 e Å^−^^3^

### Special details

**Table:**

Geometry. All esds (except the esd in the dihedral angle between two l.s. planes) are estimated using the full covariance matrix. The cell esds are taken into account individually in the estimation of esds in distances, angles and torsion angles; correlations between esds in cell parameters are only used when they are defined by crystal symmetry. An approximate (isotropic) treatment of cell esds is used for estimating esds involving l.s. planes.
Refinement. Refinement of F^2^ against ALL reflections. The weighted R-factor wR and goodness of fit S are based on F^2^, conventional R-factors R are based on F, with F set to zero for negative F^2^. The threshold expression of F^2^ > 2sigma(F^2^) is used only for calculating R-factors(gt) etc. and is not relevant to the choice of reflections for refinement. R-factors based on F^2^ are statistically about twice as large as those based on F, and R- factors based on ALL data will be even larger.

### Fractional atomic coordinates and isotropic or equivalent isotropic displacement parameters (Å^2^)

**Table:**

	*x*	*y*	*z*	*U*_iso_*/*U*_eq_	
C1	0.52162 (16)	0.1474 (2)	0.74442 (6)	0.0719 (5)	
H1A	0.5798	0.0876	0.7338	0.108*	
H1B	0.4957	0.1161	0.7736	0.108*	
H1C	0.5502	0.2393	0.7473	0.108*	
N1	0.63856 (10)	0.38227 (12)	0.59060 (4)	0.0459 (3)	
O1	0.47237 (8)	0.16745 (10)	0.66460 (3)	0.0480 (3)	
C2	0.37573 (18)	0.00455 (18)	0.70716 (6)	0.0777 (6)	
H2A	0.3187	0.0050	0.6842	0.117*	
H2B	0.3436	−0.0212	0.7359	0.117*	
H2C	0.4327	−0.0603	0.6990	0.117*	
O2	0.52896 (11)	0.38533 (11)	0.67575 (4)	0.0689 (4)	
C3	0.33760 (16)	0.2516 (2)	0.72143 (7)	0.0753 (5)	
H3A	0.3711	0.3411	0.7233	0.113*	
H3B	0.3027	0.2295	0.7500	0.113*	
H3C	0.2825	0.2511	0.6977	0.113*	
O3	0.69461 (11)	0.19726 (12)	0.51907 (4)	0.0714 (4)	
C4	0.42619 (13)	0.14629 (15)	0.71095 (5)	0.0473 (4)	
O4	0.61496 (10)	0.33760 (11)	0.46780 (3)	0.0586 (3)	
C5	0.52554 (12)	0.28259 (14)	0.65316 (5)	0.0459 (3)	
O5	0.73364 (13)	0.53528 (15)	0.66616 (5)	0.0839 (4)	
H5	0.6636 (9)	0.524 (3)	0.6645 (9)	0.126*	
C6	0.58168 (13)	0.26158 (15)	0.60781 (5)	0.0515 (4)	
H6A	0.6354	0.1873	0.6107	0.062*	
H6B	0.5259	0.2333	0.5858	0.062*	
C7	0.61304 (14)	0.41188 (16)	0.54343 (5)	0.0545 (4)	
H7A	0.6497	0.4972	0.5351	0.065*	
H7B	0.5332	0.4266	0.5407	0.065*	
C8	0.64704 (13)	0.30191 (16)	0.50949 (5)	0.0508 (4)	
C9	0.63142 (15)	0.24561 (18)	0.42820 (5)	0.0618 (4)	
C10	0.56917 (19)	0.1123 (2)	0.43595 (8)	0.0933 (7)	
H10A	0.6062	0.0599	0.4592	0.140*	
H10B	0.5677	0.0601	0.4082	0.140*	
H10C	0.4941	0.1320	0.4454	0.140*	
C11	0.75478 (18)	0.2232 (3)	0.42041 (7)	0.0882 (6)	
H11A	0.7921	0.3105	0.4192	0.132*	
H11B	0.7656	0.1754	0.3921	0.132*	
H11C	0.7850	0.1694	0.4449	0.132*	
C12	0.5793 (2)	0.3278 (2)	0.38978 (6)	0.0994 (8)	
H12A	0.5028	0.3477	0.3971	0.149*	
H12B	0.5824	0.2753	0.3621	0.149*	
H12C	0.6195	0.4123	0.3858	0.149*	
C13	0.75774 (14)	0.38686 (19)	0.60054 (5)	0.0614 (4)	
H13A	0.7921	0.4587	0.5824	0.074*	
H13B	0.7913	0.2999	0.5919	0.074*	
C14	0.78138 (16)	0.4137 (2)	0.65010 (6)	0.0750 (5)	
H14A	0.7532	0.3373	0.6680	0.090*	
H14B	0.8616	0.4180	0.6545	0.090*	

### Atomic displacement parameters (Å^2^)

**Table:**

	*U*^11^	*U*^22^	*U*^33^	*U*^12^	*U*^13^	*U*^23^
C1	0.0772 (12)	0.0836 (12)	0.0549 (10)	−0.0102 (10)	−0.0087 (9)	0.0146 (9)
N1	0.0527 (7)	0.0491 (7)	0.0360 (6)	−0.0043 (6)	0.0032 (5)	0.0030 (5)
O1	0.0588 (6)	0.0447 (5)	0.0406 (6)	−0.0079 (5)	0.0082 (4)	−0.0007 (4)
C2	0.1038 (14)	0.0652 (11)	0.0641 (11)	−0.0327 (11)	0.0156 (10)	0.0029 (9)
O2	0.1028 (10)	0.0468 (6)	0.0571 (7)	−0.0129 (6)	0.0304 (6)	−0.0092 (5)
C3	0.0693 (11)	0.0845 (13)	0.0722 (12)	0.0094 (10)	0.0238 (10)	0.0024 (10)
O3	0.0984 (9)	0.0681 (7)	0.0478 (7)	0.0330 (7)	−0.0009 (6)	−0.0002 (5)
C4	0.0546 (8)	0.0479 (8)	0.0393 (8)	−0.0062 (7)	0.0076 (7)	0.0034 (6)
O4	0.0760 (7)	0.0625 (7)	0.0374 (6)	0.0156 (6)	−0.0023 (5)	−0.0022 (5)
C5	0.0531 (8)	0.0434 (8)	0.0411 (8)	−0.0006 (7)	0.0041 (7)	0.0015 (6)
O5	0.1030 (10)	0.0812 (9)	0.0674 (8)	−0.0272 (9)	0.0050 (8)	−0.0216 (7)
C6	0.0611 (9)	0.0503 (8)	0.0430 (8)	−0.0069 (7)	0.0096 (7)	−0.0046 (7)
C7	0.0708 (10)	0.0530 (8)	0.0396 (8)	0.0118 (8)	0.0043 (7)	0.0023 (7)
C8	0.0577 (9)	0.0556 (9)	0.0391 (8)	0.0082 (8)	0.0040 (7)	0.0024 (7)
C9	0.0757 (11)	0.0699 (11)	0.0397 (9)	0.0100 (9)	−0.0004 (8)	−0.0105 (8)
C10	0.1030 (16)	0.0898 (15)	0.0872 (16)	−0.0163 (13)	−0.0079 (13)	−0.0199 (12)
C11	0.0809 (13)	0.1181 (17)	0.0658 (12)	0.0111 (13)	0.0199 (11)	−0.0130 (12)
C12	0.146 (2)	0.1065 (17)	0.0456 (11)	0.0351 (15)	−0.0202 (12)	−0.0077 (11)
C13	0.0567 (10)	0.0692 (11)	0.0584 (10)	−0.0081 (8)	0.0033 (8)	−0.0044 (8)
C14	0.0693 (12)	0.0905 (14)	0.0653 (12)	−0.0131 (10)	−0.0139 (9)	−0.0023 (11)

### Geometric parameters (Å, °)

**Table:**

C1—C4	1.507 (2)	O5—H5	0.848 (10)
C1—H1A	0.9600	C6—H6A	0.9700
C1—H1B	0.9600	C6—H6B	0.9700
C1—H1C	0.9600	C7—C8	1.517 (2)
N1—C7	1.4465 (18)	C7—H7A	0.9700
N1—C6	1.4475 (18)	C7—H7B	0.9700
N1—C13	1.458 (2)	C9—C11	1.511 (3)
O1—C5	1.3306 (17)	C9—C10	1.512 (3)
O1—C4	1.4830 (16)	C9—C12	1.516 (2)
C2—C4	1.508 (2)	C10—H10A	0.9600
C2—H2A	0.9600	C10—H10B	0.9600
C2—H2B	0.9600	C10—H10C	0.9600
C2—H2C	0.9600	C11—H11A	0.9600
O2—C5	1.1992 (17)	C11—H11B	0.9600
C3—C4	1.506 (2)	C11—H11C	0.9600
C3—H3A	0.9600	C12—H12A	0.9600
C3—H3B	0.9600	C12—H12B	0.9600
C3—H3C	0.9600	C12—H12C	0.9600
O3—C8	1.1992 (17)	C13—C14	1.504 (2)
O4—C8	1.3285 (17)	C13—H13A	0.9700
O4—C9	1.4794 (18)	C13—H13B	0.9700
C5—C6	1.505 (2)	C14—H14A	0.9700
O5—C14	1.394 (2)	C14—H14B	0.9700
			
C4—C1—H1A	109.5	N1—C7—H7B	108.4
C4—C1—H1B	109.5	C8—C7—H7B	108.4
H1A—C1—H1B	109.5	H7A—C7—H7B	107.4
C4—C1—H1C	109.5	O3—C8—O4	125.07 (14)
H1A—C1—H1C	109.5	O3—C8—C7	124.84 (14)
H1B—C1—H1C	109.5	O4—C8—C7	110.08 (13)
C7—N1—C6	113.32 (12)	O4—C9—C11	109.66 (14)
C7—N1—C13	113.11 (12)	O4—C9—C10	109.51 (14)
C6—N1—C13	114.57 (13)	C11—C9—C10	112.41 (17)
C5—O1—C4	121.77 (11)	O4—C9—C12	102.18 (14)
C4—C2—H2A	109.5	C11—C9—C12	111.49 (17)
C4—C2—H2B	109.5	C10—C9—C12	111.10 (17)
H2A—C2—H2B	109.5	C9—C10—H10A	109.5
C4—C2—H2C	109.5	C9—C10—H10B	109.5
H2A—C2—H2C	109.5	H10A—C10—H10B	109.5
H2B—C2—H2C	109.5	C9—C10—H10C	109.5
C4—C3—H3A	109.5	H10A—C10—H10C	109.5
C4—C3—H3B	109.5	H10B—C10—H10C	109.5
H3A—C3—H3B	109.5	C9—C11—H11A	109.5
C4—C3—H3C	109.5	C9—C11—H11B	109.5
H3A—C3—H3C	109.5	H11A—C11—H11B	109.5
H3B—C3—H3C	109.5	C9—C11—H11C	109.5
O1—C4—C3	110.86 (12)	H11A—C11—H11C	109.5
O1—C4—C1	108.31 (12)	H11B—C11—H11C	109.5
C3—C4—C1	113.35 (15)	C9—C12—H12A	109.5
O1—C4—C2	102.02 (12)	C9—C12—H12B	109.5
C3—C4—C2	110.68 (15)	H12A—C12—H12B	109.5
C1—C4—C2	111.02 (14)	C9—C12—H12C	109.5
C8—O4—C9	121.82 (12)	H12A—C12—H12C	109.5
O2—C5—O1	125.25 (13)	H12B—C12—H12C	109.5
O2—C5—C6	125.91 (13)	N1—C13—C14	112.53 (14)
O1—C5—C6	108.83 (12)	N1—C13—H13A	109.1
C14—O5—H5	106.1 (19)	C14—C13—H13A	109.1
N1—C6—C5	114.14 (12)	N1—C13—H13B	109.1
N1—C6—H6A	108.7	C14—C13—H13B	109.1
C5—C6—H6A	108.7	H13A—C13—H13B	107.8
N1—C6—H6B	108.7	O5—C14—C13	113.37 (16)
C5—C6—H6B	108.7	O5—C14—H14A	108.9
H6A—C6—H6B	107.6	C13—C14—H14A	108.9
N1—C7—C8	115.57 (13)	O5—C14—H14B	108.9
N1—C7—H7A	108.4	C13—C14—H14B	108.9
C8—C7—H7A	108.4	H14A—C14—H14B	107.7
			
C5—O1—C4—C3	62.24 (18)	C9—O4—C8—O3	2.7 (2)
C5—O1—C4—C1	−62.70 (17)	C9—O4—C8—C7	−176.42 (14)
C5—O1—C4—C2	−179.88 (14)	N1—C7—C8—O3	−1.9 (2)
C4—O1—C5—O2	−10.4 (2)	N1—C7—C8—O4	177.19 (13)
C4—O1—C5—C6	168.74 (12)	C8—O4—C9—C11	−63.4 (2)
C7—N1—C6—C5	−132.21 (13)	C8—O4—C9—C10	60.4 (2)
C13—N1—C6—C5	95.96 (16)	C8—O4—C9—C12	178.25 (16)
O2—C5—C6—N1	−3.5 (2)	C7—N1—C13—C14	156.64 (14)
O1—C5—C6—N1	177.39 (12)	C6—N1—C13—C14	−71.43 (18)
C6—N1—C7—C8	−62.21 (18)	N1—C13—C14—O5	−57.2 (2)
C13—N1—C7—C8	70.33 (18)		

### Hydrogen-bond geometry (Å, °)

**Table:**

*D*—H···*A*	*D*—H	H···*A*	*D*···*A*	*D*—H···*A*
O5—H5···O2	0.85 (1)	2.13 (2)	2.8658 (18)	145 (2)
